# The Effects of Royal Jelly on Fitness Traits and Gene Expression in *Drosophila melanogaster*

**DOI:** 10.1371/journal.pone.0134612

**Published:** 2015-07-30

**Authors:** John R. Shorter, Matthew Geisz, Ergi Özsoy, Michael M. Magwire, Mary Anna Carbone, Trudy F. C. Mackay

**Affiliations:** 1 Department of Biological Sciences, North Carolina State University, Raleigh, North Carolina, United States of America; 2 W. M. Keck Center for Behavioral Biology, North Carolina State University, Raleigh, North Carolina, United States of America; 3 Program in Genetics, North Carolina State University, Raleigh, North Carolina, United States of America; 4 Hacettepe University, Department of Biology, Ankara, Turkey; University of Arkansas, UNITED STATES

## Abstract

Royal Jelly (RJ) is a product made by honey bee workers and is required for queen differentiation and accompanying changes in queen body size, development time, lifespan and reproductive output relative to workers. Previous studies have reported similar changes in *Drosophila melanogaster* in response to RJ. Here, we quantified viability, development time, body size, productivity, lifespan and genome wide transcript abundance of *D*. *melanogaster* reared on standard culture medium supplemented with increasing concentrations of RJ. We found that lower concentrations of RJ do induce significant differences in body size in both sexes; higher concentrations reduce size, increase mortality, shorten lifespan and reduce productivity. Increased concentrations of RJ also consistently lengthened development time in both sexes. RJ is associated with changes in expression of 1,581 probe sets assessed using Affymetrix *Drosophila* 2.0 microarrays, which were enriched for genes associated with metabolism and amino acid degradation. The transcriptional changes are consistent with alterations in cellular processes to cope with excess nutrients provided by RJ, including biosynthesis and detoxification, which might contribute to accelerated senescence and reduced lifespan.

## Introduction

Development of female honey bee larvae into reproductive queens or non-reproductive workers is regulated by Royal Jelly (RJ), a secretion produced by worker honey bees from their mandibular and hypopharyngeal glands [1–5]. All bees are fed RJ for the first three days of their lives, but workers are switched to a worker jelly diet on the fourth day while females destined to become reproductively active are kept in specialized queen cups and fed RJ throughout their development. The physiological changes between genetically identical queens and workers are profound: in addition to ovary development and egg laying required for reproduction, queens develop faster, are larger, and live up to ten times as long as workers [6]. This combination of life history traits is remarkable because in most organisms there are negative correlations (trade-offs) between growth, reproduction and lifespan [7–12].

The profound effects of RJ on development and physiology of honey bees has stimulated many studies assessing whether its beneficial effects can transcend species barriers. RJ has been reported to increase lifespan in mice [13] and *C*. *elegans* [14]; decrease fatigue [15] in mice; increase collagen production [16], reduce hypertension [17] and modulate oxidative stress and tissue injury repair [18] in rats; slow testicular decline in hamsters [19]; inhibit the production of pro-inflammatory cytokines by activated mouse macrophages [20] and inhibit bisphenol A-induced proliferation of human breast cancer cells [21]. RJ is a popular nutritional supplement in humans [22] and has been reported to have beneficial effects on glucose tolerance, mental health, and lipoprotein metabolism [23, 24]. However, these studies in humans have small sample sizes and fail to correct statistically for multiple testing. The European Food Safety Authority does not accept claims that consuming RJ is beneficial to human health and the US Food and Drug Administration has taken legal action against companies making unfounded claims for RJ benefits to human health [25, 26]. *Drosophila melanogaster* is gaining support as a model for human health and disease [27–29] and may give insight into the mechanism(s) of action of RJ more rapidly and with less expense than vertebrate models.

The bioactive molecules in RJ and its mechanism of action in honeybees and other species are not well-understood. RJ has histone deacetylase inhibitor activity [5], and the 57-kDa royalactin protein has been reported to recapitulate the effects of RJ in honeybees [6]. RJ could exert its effects though epigenetic modifications via DNA methylation [4, 30–32] (but see Ref. [33]). Progress in identifying the bioactive molecules in and mechanisms of action of RJ would be facilitated if its effects in honey bee queens could be mimicked in the genetically tractable *Drosophila melanogaster* model. Indeed, both RJ and royalactin have been reported to reduce development time and increase body size, fecundity and lifespan in female, but not male, *D*. *melanogaster*, mediated in part via insulin and Egfr signaling pathways [6, 34].

Here, we sought to further characterize the effects of RJ on viability, development time, body size, lifespan, productivity and global gene expression in *D*. *melanogaster*. We were able to replicate the increase in body size in most lines, although the effects were not female-specific. However, RJ reduced fitness with respect to development time, lifespan and productivity. While we found substantial alterations in gene expression in flies reared on RJ, these did not involve the insulin and Egfr signaling pathways.

## Results and Discussion

### Effects of RJ on *D*. *melanogaster* fitness traits

**Fig 1:**
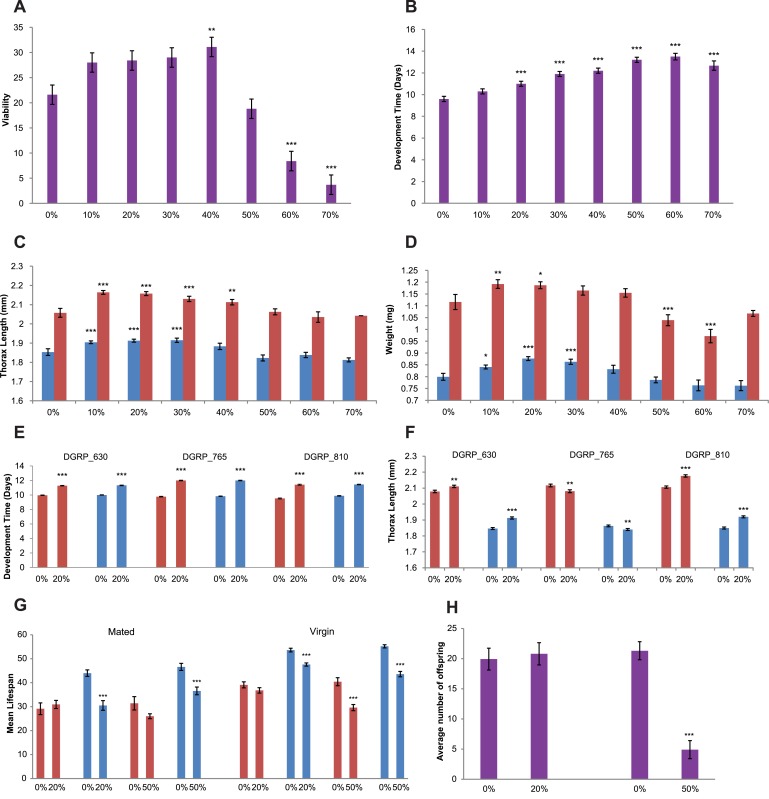
Effect of RJ on life history traits. (A) Canton S B viability. Males and females are pooled. Viability is the average number of adult flies emerging from 50 eggs. (B) Canton S B development time. Males and females are pooled. (C) Canton S B thorax length. Red bars denote females, blue bars are males. (D) Canton S B body weight. Red bars denote females, blue bars are males. (E) Development time for three DGRP lines. Red bars denote females, blue bars are males. (F) Thorax length of three DGRP lines. Red bars denote females, blue bars are males. (G) Canton S B lifespan. Red bars denote females, blue bars are males. (H) Canton S B productivity. *** *P* < 0.001, ** *P* < 0.01, * *P* < 0.05.

We first assessed viability, development time and body size on isogenic *w* ^*1118*^; Canton S B flies [35] on standard cornmeal-agar-molasses medium and medium supplemented with 10%, 20%, 30%, 40% 50%, 60% and 70% RJ. No flies survived at higher RJ concentrations than 70%. We found no significant differences in viability between 10–30% RJ, but viability significantly increased when flies were reared on 40% RJ (*P* < 0.01), and decreased at 60% and 70% RJ (*P* < 0.001) (Fig 1A). Development time, however, increases at all concentrations of RJ > 20% (*P* < 0.001) (Fig 1B). We also assessed the effects of RJ on two measures of body size: thorax length and wet weight. We found a significant increase in female thorax length at low (10–30%) concentrations of RJ (*P* < 0.01 for 30% RJ and *P* < 0.001 for 10% and 20% RJ), and for male thorax length at 20% (*P* < 0.01) and 30% (*P* < 0.001) RJ (Fig 1C). For wet weight, we observed a significant increase in males at 20% (*P* < 0.001) and 30% (*P* < 0.01) RJ, and a significant decrease in females at 60% RJ (*P* < 0.01) (Fig 1D).

To assess whether the effects we observed were strain-specific, we also quantified development time and thorax length for three *Drosophila* Genetic Reference Panel inbred lines [36, 37] reared on standard medium and medium supplemented with 20% RJ. We again found a significant increase in development time for both concentrations and both sexes for all three lines (*P* < 0.001) (Fig 1E). Supplementation with 20% RJ also affected thorax length in both sexes in the three DGRP lines, but with significant increases in two lines and significant decreases in the third (Fig 1F).

We next assessed lifespan of both virgin and mated Canton S B males and females on standard medium and medium supplemented with 20% and 50% RJ. Both concentrations of RJ significantly (*P* < 0.001) reduced male lifespan; in addition, female lifespan was reduced when reared on 50% RJ (*P* < 0.001) (Fig 1G). Lifetime productivity of females reared on 20% RJ was not altered, but was significantly (*P* < 0.001) reduced for females reared on 50% RJ (Fig 1H).

In summary, we find that RJ does affect fitness traits in *D*. *melanogaster*, but that the direction and pattern of these effects are not consistent with the decrease in development time and female-specific increase in longevity and fecundity reported previously [6, 34]. We did replicate the effect of 20% RJ increasing body size, but this effect was not female-specific, and in one of three DGRP lines tested, the effect was in the opposite direction. We also observed an increase in viability at a moderately high (40%) concentration of RJ, although even higher concentrations reduce survivorship. 20% and higher amounts of RJ increased development time in all strains tested, and reduced lifespan and productivity.

One possible explanation for the discrepancy between our results and the previous studies could be that the previous studies used a slightly different strain, Canton S. However, the Canton S B line used here is an isogenic derivative of Canton S [35] and hence related, and we replicated the development time results in three genetically distinct DGRP lines. Neither the Canton S strain nor the *UAS-Royalactin* transgenic line described previously [6] is publically available, precluding a direct replication experiment. However, our results indicate that the beneficial fitness effects of RJ reported previously are not general for all *D*. *melanogaster* genotypes. A second possible explanation is that we used a commercially available source of RJ, following the methods of others [5], and not fresh RJ [6]. Although there may be variation in the quality and composition of RJ from different sources [38].

The most likely explanation for differences between our results and those reported previously is a difference in the nutritional quality of the control culture medium. The 8% yeast, 10% D-glucose and 82% water medium used by Kamakura [6] is of poor nutritional quality, and flies reared on this medium have an unusually long development time of 12.2 days at 25°C. In contrast, the average development time of the four strains of flies used in our study, reared on standard cornmeal-agar-molasses medium, is 9.8 days. Thus, it is likely that Kamakura’s [6] control flies were under starvation stress and did not receive an optimal amount of protein. Restricting dietary protein is widely known to extend development time and reduces body size in *D*. *melanogaster* [39]. If this is the case, supplementing the control diet with RJ may have rescued the protein deficiency, resulting in an increase in all components of fitness measured [6], independent of other bioactive molecules in RJ.

### Effects of RJ on *Drosophila* gene expression

In order to assess the effect of RJ on gene expression, we reared flies on control (0% RJ) medium and medium supplemented with 20%, 50% and 60% RJ and performed genome wide expression analyses on third instar larvae and young adult males and females using Affymetrix GeneChip *Drosophila* Genome 2.0 microarrays. We used analyses of variance to partition variation in expression due to RJ treatment, developmental stage and the treatment by stage interaction for each expressed transcript. At a false discovery rate of FDR < 0.05, we found 1,581 probe sets significant for the effect of treatment, 17,833 for developmental stage and 591 for the treatment by developmental stage interaction (S1 Table). We performed Gene Ontology enrichment analysis [40, 41] for the probe sets that were significant for the effect of treatment. The top enriched GO category is oxidation reduction (S2 Table), attributable in part to genes encoding many members of the Cytochrome P450 gene family. Other enriched GO categories include genes involved in catabolism (which also contribute to the oxidation reduction GO category), metabolism and defense/immune response (S2 Table).

**Fig 2:**
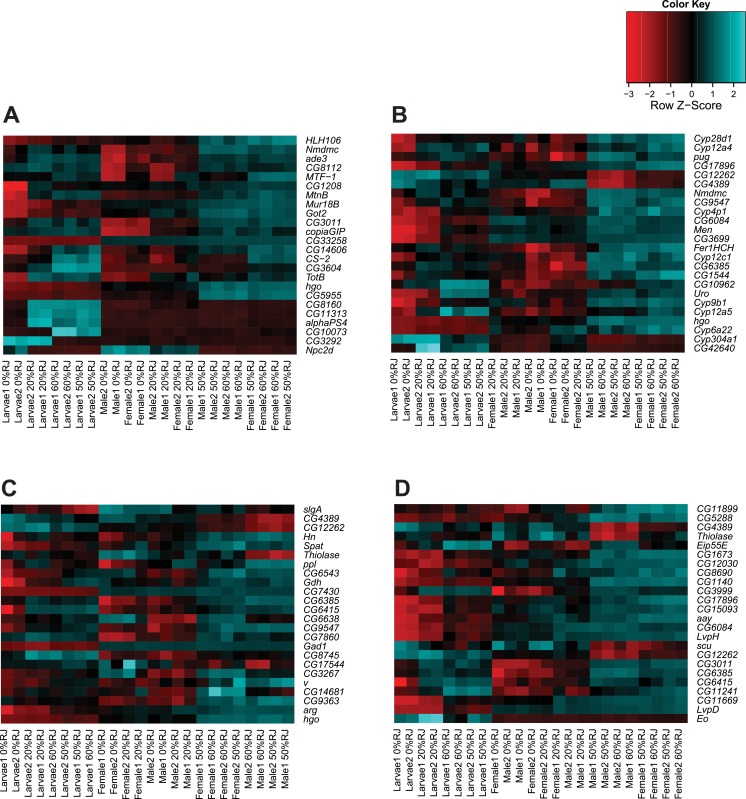
Heat maps of top differentially expressed genes across different concentrations of RJ. (A) The top 24 most differentially expressed genes (Food FDR < 8.9E-06). (B) The 24 most differentially expressed genes enriched for the ‘oxidative reduction’ GO category (FDR = 1.02E-17). (C) The 24 most differentially expressed genes enriched for the ‘carboxylic acid catabolic process’ GO category (FDR = 1.43E-09). (D) The 24 most differentially expressed genes enriched in the three KEGG pathway categories: Valine, leucine and isoleucine degradation (FDR = 1.54E-04); Galactose metabolism (FDR = 6.95E-03); Glycine, serine and threonine metabolism (FDR = 1.51E-02).

We visualized the expression patterns of the top differentially expressed genes and the top genes in the oxidative reduction and carboxylic acid catabolic process GO categories and the top genes in the three enriched KEGG pathways (valine, leucine and isoleucine degradation, galactose metabolism and glycine, serine and threonine metabolism) (Fig 2). In all cases, larval expression patterns cluster separately from those of adults, and within each developmental stage, the enriched genes are strongly upregulated in flies fed higher concentrations (50% and 60%) of RJ. These patterns are identical in adult males and females, indicating that the effects of feeding RJ on differential expression of the top genes and enriched categories are not sex-specific.

**Fig 3:**
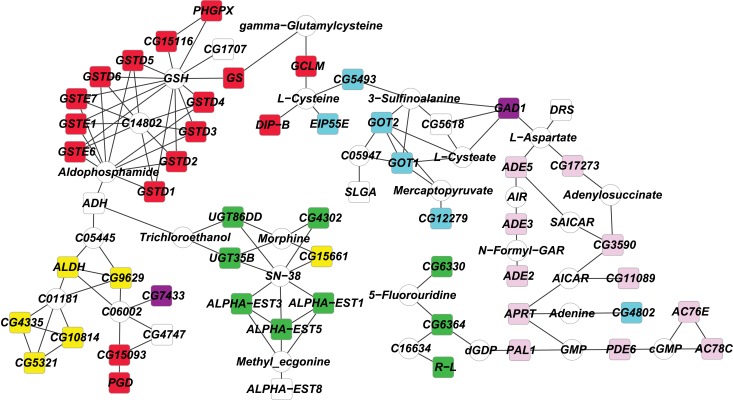
Genetic network of RJ differentially expressed genes. Colored boxes denote differentially expressed genes. Red, glutathione metabolism; pink, purine metabolism; green, drug metabolism and other enzymes; teal, cysteine and methionine metabolism; yellow, lysine degradation; purple, beta-alanine metabolism.

We used network enrichment analysis to place the differentially expressed genes significant for the treatment effect in context. We identified a genetic network of 57 genes (P < 0.01) based on global metabolic gene data [42] (Fig 3). This network is enriched for genes encoding glutathione S transferases, which detoxify xenobiotic compounds, and for genes involved in amino acid metabolism, consistent with the high levels of protein found in RJ.

In summary, we have shown that feeding high concentrations of RJ has widespread effects on *Drosophila* gene expression that are likely in part to be associated with the reduced fitness of adult flies. In contrast to previous results [6], the changes in expression of the top differentially expressed genes and GO categories are not sex-specific; neither are they enriched for insulin and Egfr signaling. RJ is enriched in protein and amino acids [38]. The enriched GO categories and genetic network are instead consistent with alterations in cellular processes to cope with these excess nutrients, including biosynthesis and detoxification, which might contribute to accelerated senescence and reduced lifespan.

## Materials and Methods

### Effects of RJ on *D*. *melanogaster* fitness traits

We obtained frozen RJ from GloryBee Foods, Eugene, Oregon. We assessed the effects on viability, development time and body size of isogenic *w* ^*1118*^ Canton S B [35] flies reared on 10 ml (0% RJ) cornmeal-agar-molasses medium (http://flystocks.bio.indiana.edu/Fly_Work/media-recipes/molassesfood.htm) and flies reared on 10 ml medium supplemented with 10%, 20%, 30%, 40%,50%, 60%, and 70% RJ by adding RJ relative to volume. The consistency of RJ is slightly more viscous than the standard culture medium. RJ has a moisture content between 53–64%, a crude protein content of 15–19.6%, a sugar content of 21.1–14.3%, with crude protein making up nearly half of the dry weight [43]. All flies were reared under standard culture conditions of 25°C, 60–75% relative humidity, 12-hr light:dark cycle. We collected eggs laid overnight on grape agar from groups of ~300 adult male and female flies, and standardized larval crowding by placing 50 eggs in each vial (*N* = 10 replicate vials per treatment). We collected emerging virgin males and females twice daily. We estimated egg-adult viability and development time as the % flies emerged in each vial and total time to eclosion, respectively. We measured the weight and thorax length of two virgin males and two virgin females from each of 10 replicate vials per treatment. To assess wet weight, we pooled the 20 males and 20 females (sexes separately), flash froze them on dry ice and weighed them immediately in groups of five using a Mettler Toledo X520514 balance. We measured thorax length laterally, from the neck to the tip of scutellum with an Olympus SZ61 dissecting microscope calibrated using a 20x/12.5 calibration ruler slide and optical micrometer. We also assessed development time and thorax length of flies from three inbred lines (DGRP_630, DGRP_765, DGRP_810) from the *Drosophila* Genetic Reference Panel chosen based on approximately similar development times [36, 37] reared on 0% and 20% RJ. DGRP lines were measured using a light microscope connected to a computer with ImageJ image processing software [44]. We quantified lifespan of mated and virgin males and females and productivity of mated females for *w* ^*1118*^ Canton S B flies reared on 0%, 20% and 50% RJ. To minimize variation of the adults placed in the experimental design, we set up 18 controlled adult density (CAD) flies with six pairs of males and females and cleared all flies from vials after four days of random mating and egg laying. Emerging virgin flies were collected from these CAD vials with concentrations of 0%, 20% and 50% RJ and measured for lifespan and total number of offspring on control food. Groups of six virgin males, six virgin females, and three virgin males and females were placed into each of 24 vials per treatment. Flies were transferred to fresh vials every two days and scored for survival until all were dead. We retained the vials with males and females after each transfer and counted the total number of emerging offspring for an individual vial for 15 days following setup. This length was selected because it allows for nearly all of the eggs in a vial to hatch but is short enough to not be contaminated by an F2 generation. We used Student’s *t*-tests to evaluate differences between RJ treatments and the control.

### Effects of RJ on *Drosophila* gene expression

We collected eggs laid overnight on grape agar from groups of ~300 adult flies, and placed 50 eggs in 4–6 replicate vials for each of four treatments: 0, 20%, 50%, and 60% RJ, with 60% RJ having 6 replicate vials due to low viability. We collected two biological replicates each with 40 third instar larvae, ten 3–5 day old virgin females and 15 3–5 day old virgin males per treatment between 8:00 am and 11:00 am across nine days and samples were randomly pooled across vials and days. The larvae and adults were frozen on dry ice and stored at -80°C. Total RNA was extracted from each sample using ceramic beads and the Qiagen miRNeasy 96 kit. Biotinylated cRNA probes were prepared from total RNA of each sample following the Affymetrix 3’-IVT Express labeling protocol. The cRNA preparations were then fragmented and hybridized to GeneChip *Drosophila* Genome 2.0 microarrays (Affymetrix inc). Intensities of PM probes were adjusted for background hybridization using GCRMA [45], and quantile normalized before they were summarized into probe set expression using median polish. All microarray preprocessing steps were carried out using R [46] and SAS Quality assessments were analyzed by visual confirmation and boxplots, with no observed aberrations between replicate probe-sets. To determine significance, we first ran ANOVAs on status (male, female, or larvae) and food type (four concentrations of RJ) and then performed a Tukey test across sex, food, status and every possible interaction. We also calculated the false discovery rate (FDR), a measure of experiment-wide, as opposed to individual-test significance, and set the threshold for significance at 0.05 using SAS software [47].

To determine gene ontology (GO) for the 1,581 probes significantly altered in expression in response food status, we used DAVID, an online functional annotation and bioinformatics tool [40, 41]. To determine if any of these genes identified were enriched in a metabolic network, we used KEGG spider, an online tool for interpretation of genetic data in the context of global gene metabolic networks [42].

## Supporting Information

S1 Table Microarray data analyses. *P*-values and false discovery rates from ANOVA of all expressed probe sets and annotations are given. Columns O-W give Tukey test results.
(XLSX)

S2 Table Gene Ontology (GO) enrichment analyses for probe sets significant (FDR < 0.05) for the Food term in ANOVA of microarray data. (A) Enrichment of individual GO terms. (B) Cluster analysis.
(XLSX)
